# The IL-17 receptor IL-17RE mediates polyIC-induced exacerbation of experimental allergic asthma

**DOI:** 10.1186/s12931-020-01434-9

**Published:** 2020-07-08

**Authors:** Giovanna Vella, Lars Lunding, Felix Ritzmann, Anja Honecker, Christian Herr, Michael Wegmann, Robert Bals, Christoph Beisswenger

**Affiliations:** 1 Department of Internal Medicine V – Pulmonology, Allergology and Critical Care Medicine, Saarland University, D-66421 Homburg, Germany; 2 Division of Asthma Exacerbation & Regulation, Priority Area Asthma and Allergy, Leibniz Lung Center Borstel, Airway Research Center North (ARCN), Member of the German Center for Lung Research (DZL), Borstel, Germany

## Abstract

**Background:**

The interleukin 17 receptor E (IL-17RE) is specific for the epithelial cytokine interleukin-17C (IL-17C). Asthma exacerbations are frequently caused by viral infections. Polyinosinic:polycytidylic acid (pIC) mimics viral infections through binding to pattern recognition receptors (e.g. TLR-3). We and others have shown that pIC induces the expression of IL-17C in airway epithelial cells. Using different mouse models, we aimed to investigate the function of IL-17RE in the development of experimental allergic asthma and acute exacerbation thereof.

**Methods:**

Wild-type (WT) and IL-17RE deficient (*Il-17re*^*−/−*^) mice were sensitized and challenged with OVA to induce allergic airway inflammation. pIC or PBS were applied intranasally when allergic airway inflammation had been established. Pulmonary expression of inflammatory mediators, numbers of inflammatory cells, and airway hyperresponsiveness (AHR) were analyzed.

**Results:**

Ablation of IL-17RE did not affect the development of OVA-induced allergic airway inflammation and AHR. pIC induced inflammation independent of IL-17RE in the absence of allergic airway inflammation. Treatment of mice with pIC exacerbated pulmonary inflammation in sensitized and OVA-challenged mice in an IL-17RE-dependent manner. The pIC-induced expression of cytokines (e.g. keratinocyte-derived chemokine (KC), granulocyte-colony stimulating factor (G-CSF)) and recruitment of neutrophils were decreased in *Il-17re*^*−/−*^ mice. pIC-exacerbated AHR was partially decreased in *Il-17re*^*−/−*^ mice.

**Conclusions:**

Our results indicate that IL-17RE mediates virus-triggered exacerbations but does not have a function in the development of allergic lung disease.

## Background

Asthma exacerbations cause considerable morbidity and are frequently associated with rhinovirus and respiratory syncytial virus infections [1, 2]. The two-hit hypothesis states that viral infections represent a second hit triggering acute asthma exacerbation in patients suffering from already established allergic lung inflammation as a first hit [3]. There is evidence that viral RNAs cause exacerbation-associated inflammation and that dsRNA motifs (e.g. polyinosinic:polycytidylic acid (pIC)) trigger exacerbation similar to rhinovirus infections in models of experimental asthma [4–6].

The IL-17 receptor family consists of five receptor subtypes (IL-17RA to IL-RE), which interact with different members of the IL-17 cytokine family (Il-17A to F) [7, 8]. IL-17C is suggested to signal through a complex of IL-17RE and IL-17RA, whereas IL-17RA is also forming a heterodimeric receptor complex with IL-17RC for IL-17A signaling [8]. IL-17RE is primarily expressed by epithelial cells and lymphocytes, such a Th17 cells, whereas IL-17RA is ubiquitously expressed [9–13].

There is a functional overlap between IL-17A and IL-17C. Both cytokines mediate the expression of cytokines, chemokines, and antimicrobial peptides [8]. However, IL-17A is expressed by immune cells (e.g. Th17 cells, tissue resident T cells), whereas IL-17C is mainly of epithelial origin [8, 9, 12–14]. In vitro and in vivo studies showed that the expression of IL-17C in airway epithelial cells is induced by lung pathogens including rhinoviruses and that IL-17C promotes the recruitment of neutrophils into the lung [12–22].

Studies suggest a function for IL-17A and IL-17RA in the development of allergic inflammation of the lung and airway hyper-responsiveness (AHR) [5, 23–26]. It has been demonstrated that IL-17A promotes contractile force generation of airway smooth muscle through IL-17RA [23, 24]. Because of the functional overlap between IL-17A and IL-17C and the corresponding receptor complexes IL-17RA/IL-17RC and IL-17RA/IL-17RE, we examined the function of IL-17RE in mouse models of OVA-induced experimental asthma and acute exacerbation thereof. We provide evidence that IL-17RE does not have a function in the development of allergic airway inflammation and AHR. However, our data indicate that IL-17RE contributes to pIC-triggered exacerbation once allergic airway inflammation has been established.

## Material and methods

### Mice

IL-17RE-deficient (*Il-17re*^*−/−*^) C57BL/6 mice were initially obtained from Mutant Mouse Resource and Research Center (MMRRC, USA). Female mice *Il-17re*^*−/−*^ mice and their wild-type (WT) littermates were used at the age of 9–11 weeks. Breeding of animals and all animal experiments were approved by the Landesamt für Soziales, Gesundheit und Verbraucherschutz of the State of Saarland and by the animal ethics committee from the Department of State, Kiel, Germany. All experiments were done under consideration of the national guidelines for animal treatment.

### Experimental protocol

WT and *Il-17re*^*−/−*^ mice were sensitized by i.p. injection with aluminum-hydroxide-adsorbed OVA (2 mg aluminum hydroxide (ThermoFisher, Waltham, USA) with 20 μg ovalbumin (Sigma-Aldrich, St. Louis, USA)) on days 1, 14, and 21. To induce acute allergic airway inflammation mice were exposed three times to an OVA aerosol (1% OVA in PBS) on days 26, 27, and 28. Control mice received PBS (i.p.) and were challenged with OVA aerosol. Mice were treated with pIC as described previously [5]. In brief, mice were anaesthetized by i.p. injection of ketamine (105 mg/kg body weight, Bayer, Leverkusen, Germany) and xyalizine (7 mg/kg body weight, Serumwerk Bernburg AG, Bernburg, Germany) 2 h after the final OVA challenge. 100 μg pIC (Sigma-Aldrich, St. Louis, USA) dissolved in 20 μl sterile PBS or 20 μl PBS without pIC were administrated intranasally.

### Bronchoalveolar lavage and cytokine measurements

Bronchoalveolar lavage (BAL) fluids were collected 24 h after the final OVA challenge as described before [18, 21]. In brief, mice were euthanized, the tracheae were cannulated and BAL was performed with 1 ml of PBS flushed three times into the lungs. Numbers of immune cells were counted by using a hemocytometer (Innovatis AG, Reutlingen, Germany). Leukocytes were differentiated by DiffQuick Staining (Medion Diagnostics, Miami, FL, USA) on cytospin preparations. Lavaged lungs were homogenated in 1 ml PBS. BAL fluids and lung homogenates were centrifuged and the supernatants were kept at − 80 °C. Cytokines were measured by ELISA (R&D, Minneapolis, MN, USA).

### Quantitative RT-PCR

RNA was isolated from lung tissue with Trizol Reagent (Life Technologies, USA) 4 h after treatment with pIC or PBS according to the manufacturer’s protocol. 2 μg of total RNA was used for the synthesis of cDNA and real-time PCR were performed as described before [17, 18, 27, 28].

### Lung histology

Immunohistochemical staining was done as described before [17, 21]. In brief, lungs were fixed by instillation of 4% formalin buffered in PBS under a constant hydrostatic pressure (30 cm H_2_O for 15 min), embedded in 1% agarose, cut into slices of exactly the same thickness, and embedded in paraffin. Samples were permeabilized with 0.5% Tween-20. Following primary antibodies were used: CD4 (ab183685, 1/100; Abcam, Cambridge, UK) and Ly6B (MCA771GA, 1/150; Bio-Rad, Munich, Germany). HRP-conjugated secondary antibodies (Histofine Simple Stain, Nichirei Biosciences Inc. Japan) were used. Randomly selected fields were evaluated for positive cells blinded to the investigator using the Visiopharm Integrator System (Visiopharm, Hoersholm, Denmark) on an Olympus BX51 microscope.

### Lung function

Airway reactivity was assessed by methacholine (MCh, acetyl-b-methylcholine chloride; Sigma-Aldrich, St. Louis, MO, USA) provocation and recorded using invasive lung function assessment (FinePointe RC units; Data Sciences International, New Brighton, MN, USA) as described before [5]. Mice were anesthetized with ketamine (90 mg/kg body weight; cp-pharma, Burgdorf, Germany) and xylazine (10 mg/kg; cp-pharma), cannulated, and mechanically ventilated with 150 ml/breath. Mice were provoked with increasing concentrations of MCh aerosols as indicated. Each aerosol provocation lasted for 30 s and was followed by 270s incubation time.

### Statistical analysis

Comparisons were tested with two-way ANOVA (Tukey post-test) using the software Prism (GraphPad Software, San Diego, CA). The results were considered statistically significant for *P* < 0.05.

## Results

### pIC induces lung inflammation in the absence of experimental allergic asthma independent of IL-17RE

**Fig. 1:**
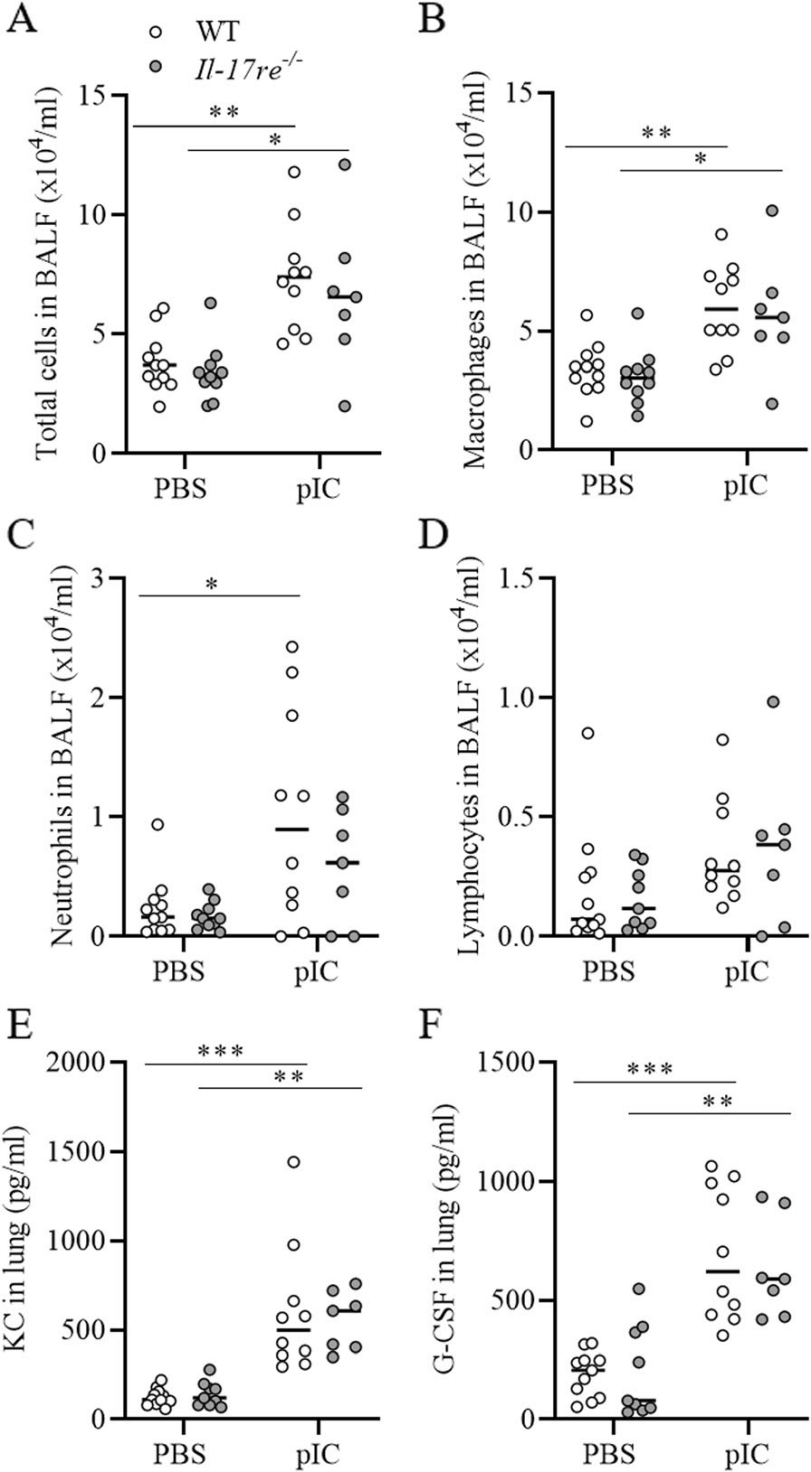
IL-17RE does not mediate pIC-induced lung inflammation in the absence of experimental allergic asthma. WT and *Il-17re*^*−/−*^ mice were intranasally challenged with 100 μg pIC or PBS as control and analyzed after 24 h. Numbers of total cells (**a**), macrophages (**b**), neutrophils (**c**), and lymphocytes (**d**) were determined in BAL fluids. Concentrations of KC (**e**) and G-CSF (**f**) were measured in lung tissue. Data were compared by Two-way ANOVA with Tukey’s post-test and are shown as the mean ± SEM. *p < 0.05, **p < 0.01, and ***p < 0.001

First, we studied the function of IL-17RE in pIC-induced lung inflammation. Therefore, we intranasally treated WT and *Il-17re*^*−/−*^ mice with pIC or PBS as control. Administration of pIC resulted in significantly increased numbers of total cells in BAL fluids independent of IL-17RE (Fig. 1a). Differential cell counting showed no significant difference in the numbers of macrophages, neutrophils, and lymphocytes between WT and *Il-17re*^*−/−*^ mice 24 h after pIC challenge (Fig. 1b-d). Moreover, treatment with pIC resulted in significantly increased concentrations of KC and G-CSF in lungs of both, WT and *Il-17re*^*−/−*^ mice (Fig. 1e and f).

### IL-17RE mediates pIC-induced lung inflammation

**Fig. 2:**
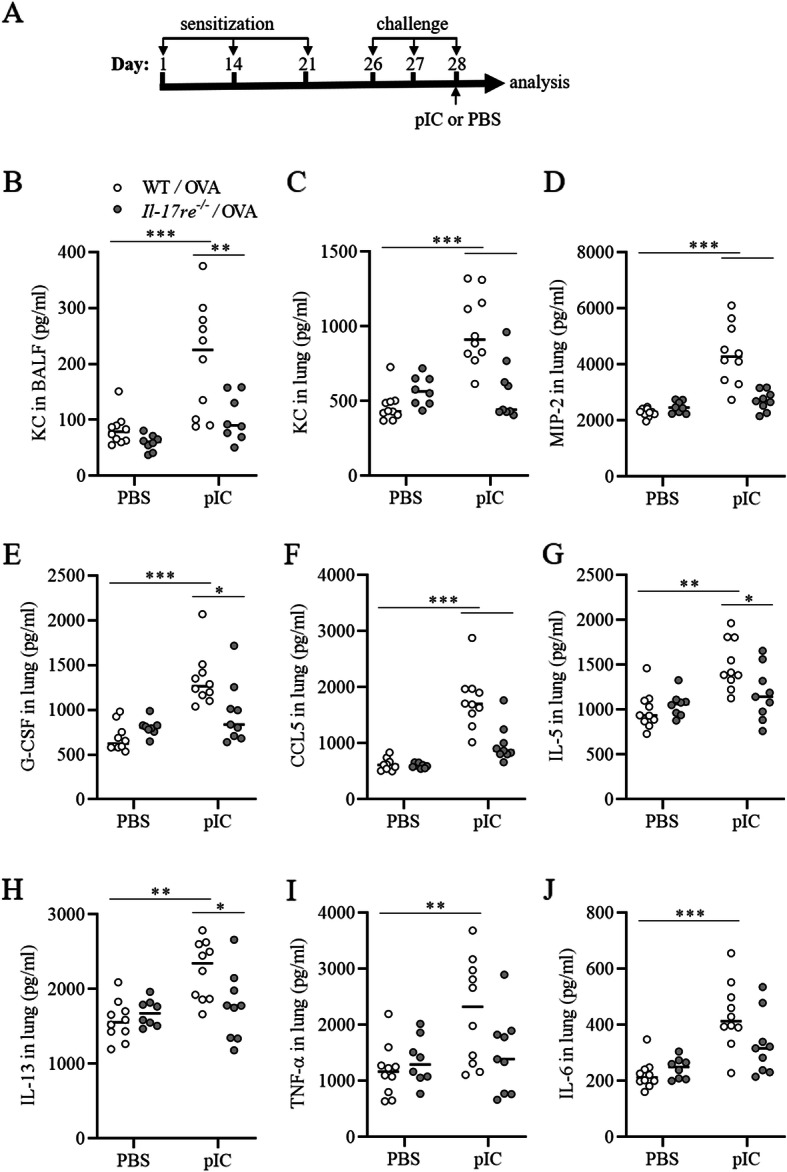
pIC-exacerbated lung inflammation depends on IL-17RE. OVA-sensitized and challenged WT and *Il-17re*^*−/−*^ mice were intranasally treated with 100 μg pIC or PBS as control. **a** Schema of the experimental protocol to study pIC-exacerbated OVA-induced allergic lung inflammation. Mice were treated with pIC or PBS as control 2 h after the final OVA challenge. BAL fluid concentrations of KC (**b**) and lung tissue concentrations of KC (**c**), MIP-2 (**d**), G-CSF (**e**), CCL5 (**f**), IL-5 (**g**), IL-13 (**h**), TNF-α (**i**), and IL-6 (**j**) were measured 22 h after treatment with pIC or PBS. Data were compared by Two-way ANOVA with Tukey’s post-test and are shown as the mean ± SEM. *p < 0.05, **p < 0.01, and ***p < 0.001

Recent studies indicate a function for IL-17A/F and the IL-17 receptors IL-17RA and IL-17RC in models of experimental asthma [5, 24, 29]. As the function of IL-17RE in the development of allergic airway inflammation is unknown we subjected WT mice and *Il-17re*^*−/−*^ littermates to a well-established mouse model of experimental allergic asthma [5, 30, 31]. In order to mimic viral infection, we applied pIC intranasally 2 h after the final OVA challenge and, thus, at a time point when allergic lung inflammation had already been established and analyzed the animals 22 h later (Fig. 2a).

**Fig. 3:**
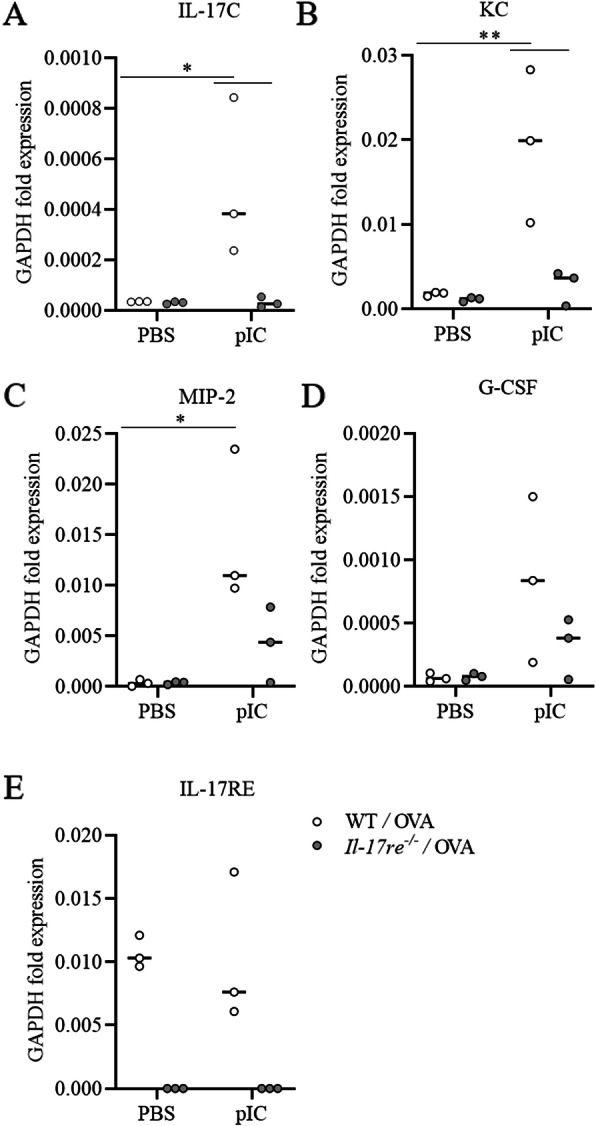
IL-17RE regulates the pIC-induced expression of cytokines in asthmatic mice. Asthmatic WT and *Il-17re*^*−/−*^ mice were intranasally treated with 100 μg pIC or PBS as control 2 h after the final OVA challenge and analyzed after 4 h. The lung tissue mRNA expression of IL-17C (**a**), KC (**b**), MIP-2 (**c**), G-CSF (**d**), and IL-17RE (**e**) were measure by semi-quantitative RT-PCR. Data were compared by Two-way ANOVA with Tukey’s post-test and are shown as the mean ± SEM. *p < 0.05 and **p < 0.01

**Fig. 4:**
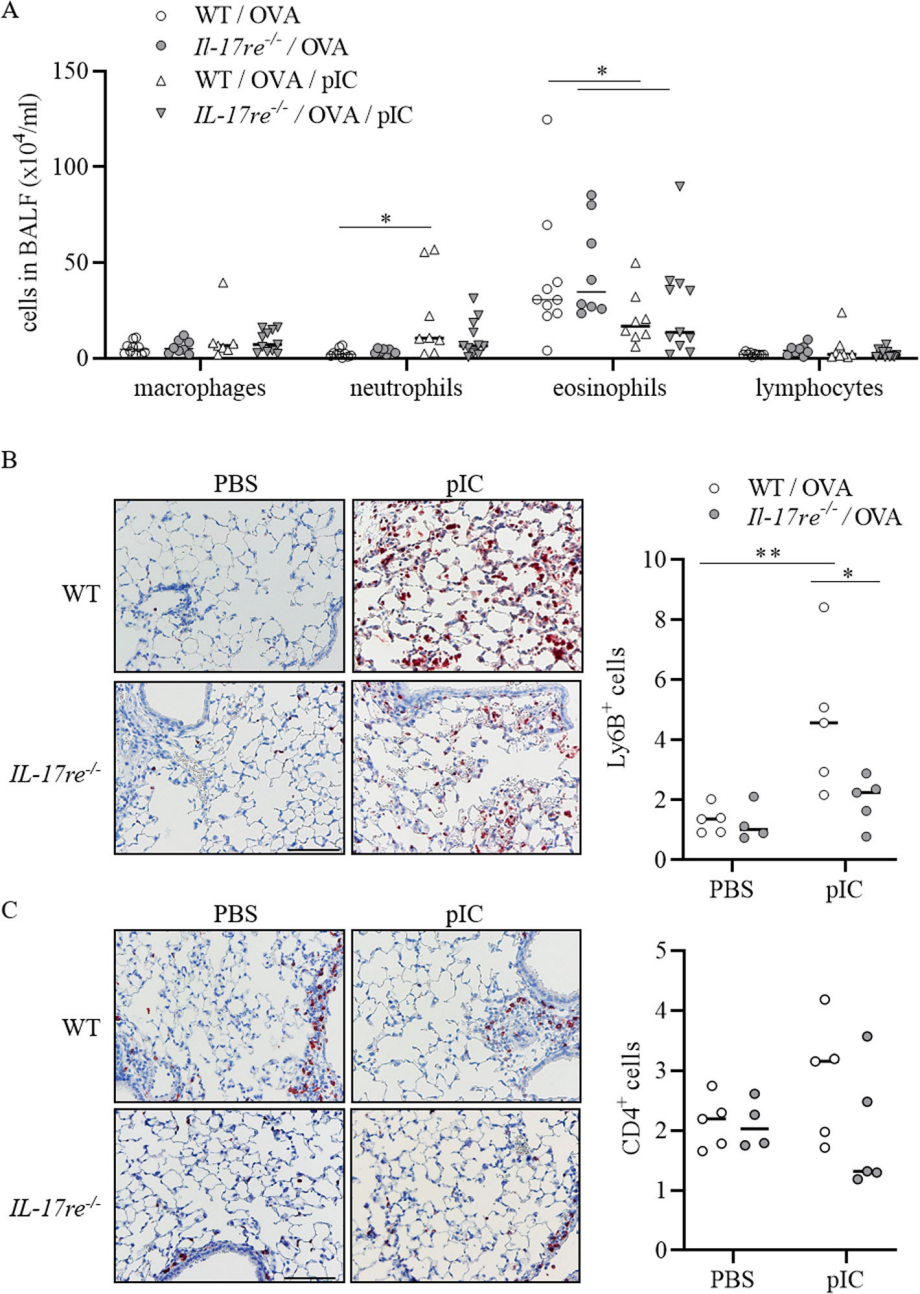
IL-17RE deficiency affects numbers of inflammatory cells in lung tissue. Asthmatic WT and *Il-17re*^*−/−*^ mice were intranasally treated with 100 μg pIC or PBS as control 2 h after the final OVA challenge and analyzed after 22 h. **a** Numbers of immune cells in BAL fluids. **b** Representative IHC of Ly6B (**b**) and CD4 (**c**) and quantification of Ly6B^+^ and CD4^+^ cells. Data were compared by Two-way ANOVA with Tukey’s post-test and are shown as the mean ± SEM. *p < 0.05 and **p < 0.01

IL-17RE deficiency did not impact OVA-induced allergic airway inflammation. There was no significant difference in the amounts of the measured pulmonary cytokines and chemokines (Figs. 2 and 3) and numbers of immune cells (Fig. 4) between PBS-treated asthmatic WT and *Il-17re*^*−/−*^ mice. However, treatment with pIC resulted in significantly increased pulmonary concentrations of the chemokines KC, MIP-2, G-CSF, and CCL5 and the cytokines IL-5 and IL-13 in WT mice compared to pIC-treated *Il-17re*^*−/−*^ mice and PBS-treated control mice (Fig. 2b-h). Moreover, administration of pIC did not result in significantly increased pulmonary concentrations of all measured cytokines in *Il-17re*^*−/−*^ mice compared to *Il-17re*^*−/−*^ control mice (Fig. 2b-j). We further determined the lung tissue expression of cytokines via semi-quantitative RT-PCR 4 h after treatment with pIC (Fig. 3a-d). pIC-induced a significantly increased expression of IL-17C, KC, and MIP-2 in WT mice, but not in *Il-17re*^*−/−*^ mice. The relative expression of IL-17RE was not affected by pIC and IL-17RE was not detectable in *Il-17re*^*−/−*^ mice verifying the expected result (Fig. 3e).

Treatment with pIC resulted in significantly increased numbers of neutrophils in BAL fluids only in WT mice, whereas numbers of eosinophils were significantly reduced in WT and *Il-17re*^*−/−*^ mice (Fig. 4a). We further analyzed the expression of Ly6B, a marker for neutrophils and inflammatory monocytes [32], in lungs of asthmatic mice not subjected to BAL by immunohistochemistry (IHC). Treatment with pIC resulted in significantly increased numbers of Ly6B^+^ cells in WT mice compared to pIC-treated *Il-17re*^*−/−*^ mice and PBS-treated control mice (Fig. 4b). IHC further showed that treatment with pIC resulted in slightly, but not significantly increased numbers of CD4^+^ in WT mice (Fig. 4c).

### IL-17RE contributes to airway hyperresponsiveness

**Fig. 5:**
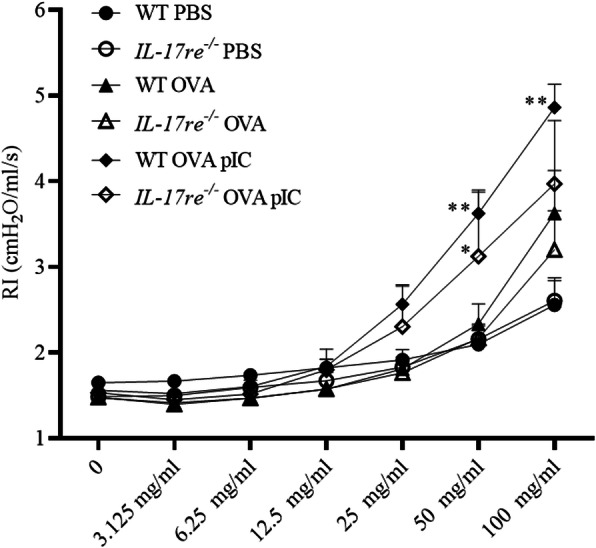
IL-17RE deficiency effects methacholine-induced AHR. OVA-sensitized and challenged WT and *Il-17re*^*−/−*^ mice were intranasally treated with 100 μg pIC or PBS as control 2 h after the final OVA challenge and analyzed after 22 h. Non-sensitized (PBS) control mice were treated with PBS. Mice were provoked with increasing concentrations of methacholine and the airway responsiveness was measured. Data were compared by Two-way ANOVA with Tukey’s post-test and are shown as the mean ± SEM. **p < 0.01 indicate a significant difference compared to corresponding OVA-sensitized and challenged control mice. *n* = 6–7 per group

We further examined the role of IL-17RE in AHR development using MCh-provocation tested 22 h after treatment with pIC. There was no significant difference in the AHR between asthmatic WT and *Il-17re*^*−/−*^ mice (Fig. 5). Treatment with pIC resulted in an exacerbated AHR in WT mice and to a lesser degree in *Il-17re*^*−/−*^ mice. The airway resistance was significantly increased in response to 50 and 100 mg/ml MCh in WT mice, whereas, in *Il-17re*^*−/−*^ mice, the pIC-induced increase in AHR reached statistical significance only at a concentration of 50 mg/ml methacholine compared to asthmatic control mice. However, the difference in the AHR did not reach statistical significance between pIC-treated WT and *Il-17re*^*−/−*^ mice after pIC treatment.

## Discussion

In this study, we examined the function of the IL-17 receptor IL-17RE in pIC-triggered exacerbation of experimental asthma. The main findings are: (1) IL-17RE is not required for the development of OVA-induced lung inflammation and AHR; (2) IL-17RE does not mediate pIC-induced pulmonary inflammation in the absence of allergic inflammation; (3) IL-17RE promotes pIC-triggered inflammation once allergic airway inflammation has established.

Preclinical studies showed that IL-17A and IL-17RA mediate the development of allergic inflammation of the airways and AHR through the recruitment of inflammatory cells and the induction of smooth muscle contraction (22–25). As it has been shown that IL-17A and IL-17C both require IL-17RA for the induction of inflammatory mediators in target cells and IL-17C promotes Th17 cell responses [11, 33, 34] we hypothesized that the specific IL-17C receptor IL-17RE has a function in OVA-induced experimental asthma. However, we did not detect any significant difference in the amounts of pulmonary cytokines, numbers of pulmonary immune cells, and AHR between WT mice and *Il-17re*^*−/−*^ littermates in the presence or absence of allergic lung inflammation. Therefore, our data do not indicate that IL-17RE has a role in the development of OVA-induced allergic lung inflammation and AHR. This finding can be explained by the relatively low expression of the IL-17RE ligand IL-17C in the absence of additional stimuli, such as ligands for pattern recognition receptors, the suppressive effect of Th2 cytokines on IL-17C expression, and a possible lack of IL-17RE expression in airway smooth muscles [12, 16, 35].

As intranasal inoculation with pIC mimics viral infections we studied the function of IL-17RE in pIC-triggered lung inflammation [4–6]. In our experimental setup, intranasal application of pIC resulted in a moderate lung inflammation, which was slightly, but not significantly decreased in *Il-17re*^*−/−*^ mice. However, once allergic inflammation had been established, pIC-triggered lung inflammation in an IL-17RE-depended manner. Pulmonary concentrations of inflammatory cytokines, numbers of neutrophils in BAL fluids, and numbers of Ly6B^+^ in the lung parenchyma were decreased in *Il-17re*^*−/−*^ mice. Thus, IL-17RE promotes inflammation in an already diseased lung.

There is evidence that IL-17RE meditates T cell activation, including the expression of effector cytokines (e.g. IL-17A) [11, 33, 36]. It has been shown that IL-17RE is highly expressed in Th17 cells and that the IL-17C/IL-17RE-axis enhances the expression of cytokine by effector Th17 cells in a in a model of autoimmune disease [11]. IL-17RE is also highly expressed by liver resident CD4^+^ T cells and natural killer T cells and augments T cell function in autoimmune hepatitis together with IL-17C [37]. Deficiency of IL-17RE also provided protection in a model of crescentic nephrotoxic nephritis, which was associated with a reduced Th17 response [33]. Thus, increased expression of inflammatory mediators by CD4^+^ and natural killer T cells already present in asthmatic mice could be a mechanism by which IL-17RE increases pulmonary inflammation in pIC-treated mice.

Ablation of IL-17RE resulted in a partial reduction of AHR in pIC-treated mice. It has been shown that neutrophil depletion partially decreases AHR in experimental asthma [23]. In addition, Toussaint et al. showed that DNA released by neutrophils promotes rhinovirus-induced type-2 allergic asthma exacerbation [38]. Thus, in our experimental setup, pulmonary neutrophils may affect MCh-induced AHR, whereas IL-17RE does not seem to have a direct function in the induction of smooth muscle contraction.

## Conclusion

In summary, our findings suggest that IL-17RE does not have a function in the development of allergic lung disease. However, once allergic inflammation has been established, IL-17RE mediates virus-triggered lung inflammation.

## Data Availability

The datasets generated and analyzed during the current study are available from the corresponding author on reasonable request.

## Abbreviations

TLR: Toll-Like Receptor
pIC: polyinosinic:polycytidylic acid
IL-17RE: Interleukin 17 receptor E
IL-17C: Interleukin-17C
WT: Wild-type
AHR: Airway hyperresponsiveness
KC: Keratinocyte-derived chemokine
G-CSF: Granulocyte-colony stimulating factor
ds: double-stranded
MCh: Methacholine
OVA: Ovalbumin
